# Graduated compression stockings in the prevention of postoperative pulmonary embolism. A propensity-matched retrospective case-control study of 24 273 patients

**DOI:** 10.1016/j.amsu.2020.06.034

**Published:** 2020-06-30

**Authors:** Kenan Suna, Eva Herrmann, Knut Kröger, Thomas Schmandra, Elisa Müller, Ernst Hanisch, Alexander Buia

**Affiliations:** a Clinic for General, Visceral and Thoracic-Surgery, Asklepios Klinik Langen, Academic Teaching Hospital Goethe-Universität Frankfurt, Röntgenstr 20, Langen, 63225, Germany; b Institute of Biostatistics und Mathematical Modeling, Klinikum und Fachbereich Medizin der Goethe-Universität Frankfurt, Theodor-Stern-Kai 7, Frankfurt Am Main, 60590, Germany; c Clinic for Angiology, Helios Klinikum Krefeld, Lutherplatz 40, Krefeld, 47805, Germany; d Department of Vascular Surgery, Heart and Vascular Clinic, Bad Neustadt, Salzburger Leite 1, Bad Neustadt a. d. Saale, 97616, Germany

**Keywords:** Venous thromboembolism, Pulmonary embolism, Deep vein thrombosis, Compression stocking

## Abstract

**Introduction:**

Recommendations for venous thromboembolism and deep venous thrombosis (DVT) prophylaxis using graduated compression stockings (GCS) is historically based and has been critically examined in current publications. Existing guidelines are inconclusive as to recommend the general use of GCS.

Patients/Methods: 24 273 in-patients (general surgery and orthopedic patients) undergoing surgery between 2006 and 2016 were included in a retrospectively analysis from a single center. From January 2006 to January 2011 perioperative GCS was employed additionally to drug prophylaxis and from February 2011 to March 2016 patients received drug prophylaxis alone. According to german guidelines all patients received venous thromboembolism prophylaxis with weight-adapted LMWH. Risk stratification (low risk, moderate risk, high risk) was based on the guideline of the American College of Chest Physicians. Data analysis was performed before and after propensity matching (PM). The defined primary endpoint was the incidence of symptomatic or fatal pulmonary embolism (PE). A secondary endpoint was the incidence of deep venous thromboembolism (DVT).

**Results:**

After risk stratification (low risk n = 16 483; moderate risk n = 4464; high risk n = 3326) a total of 24 273 patient were analyzed. Before to PM the relative risk for the occurrence of a PE or DVT was not increased by abstaining from GCS. After PM two groups of 11 312 patients each, one with and one without GCS application, were formed. When comparing the two groups, the relative risk (RR) for the occurrence of a pulmonary embolism was: Low Risk 0.99 [CI95% 0.998–1.000]; Moderate Risk 0.999 [CI95% 0.95–1.003]; High Risk 0.996 [CI95% 0.992–1.000] (p > 0.05). The incidence of PE in the total group LMWH alone was 0.1% (n = 16). In the total group using LMWH + GCS, the incidence was 0.3% (n = 29). RR after PM was 0.999 [CI95% 0.998–1.00].

**Conclusion:**

In comparison to prior studies with only small numbers of patients our trial shows in a large group of patients with moderate and high risk developing VTE we can support the view that abstaining from GCS-use does not increase the incidence of symptomatic or fatal PE and symptomatic DVT.

## Highlights

• The abstain of GCS did not result in higher incidence of pulmonary embolism.
• Concerning PE abstain of GCS shows no increase of relative risk in the group not using GCS vs. the group using GCS additionally to LMWH-prophylaxis.
• This effect is shown in the overall and the high risk population validating the results of prospectively randomized trials in a real world scenario.

## 1 Introduction

Pulmonary embolism (PE) is a dreaded postoperative complication, with up to 25% mortality and an estimated incidence of 75–269 cases per 100 000 persons [1,2]. Deep venous thrombosis (DVT), the prevalence of which is 15–40% for general surgery and 40–60% for orthopedic surgery (hip/knee), is, therefore, the origin for pulmonary embolism in 38–57.8% of cases [3,4]. International guidelines varyingly recommend thromboembolic prophylaxis with graduated compression stockings (GCS) in addition to drug prophylaxis (Appendix A) [[5], [6], [7], [8], [9], [10]]. This recommendation is increasingly being questioned. In 2011, Kröger et al. criticized the general recommendation for antithrombotic stockings as an additional prophylactic measure, since evidence for their use stemmed from older studies with small case numbers [11]. Since then, several investigators have shared this critical view [[12], [13], [14]]. In a multi-center study for hip replacement surgery (considered as a high risk group) with standard low molecular weight heparin (LMWH) therapy, Cohen et al. found no risk reduction for thromboembolic events with the additional use of GCS [15]. During conservative treatment after apoplexy, the CLOTS-1 trial also showed no risk reduction with the use of antithrombotic stockings [16]. Against this background our objective is analyzing GCS concomitantly to low molecular weight heparin (LMWH) prophylaxis in decreasing the incidence of postoperative pulmonary embolism.

## 2 Methods

### 2.1 Study design

The study was carried out according to the principles of the Declaration of Helsinki [17]. The protocol was approved by the Ethics Committee of the Hessian State Medical Association (FF 117-2016) and reporting is based on STROCSS 2019 guidelines (Strengthening the Reporting of cohort studies in surgery) [18]. The study is registered in the German Register of Clinical Trials (DRKS) Freiburg under DRKS00015507 (https://tinyurl.com/ybfkt759). From Jan. 1, 2006 to Mar. 31, 2016, all operated patients’ medical records (n = 24 273) were retrospectively analyzed from the Departments of Orthopedics/Traumatology and General, Visceral and Thoracic Surgery. Patients under 18 years of age were excluded from the study. From Jan. 1, 2006 to Jan. 30, 2011 all patients routinely received GCS (Appendices A + B), after excluding those patients with contraindications (peripheral arterial occlusive disease) set forth by medical and nursing standards. From Feb. 31, 2011 onwards no GCS were used in the clinic after the paper of Kröger ^11^ and executive board decision. Patients in the month of February 2011 were excluded from the analysis. Over the entire period, drug-based thrombosis prophylaxis with LMWH began on the day of admission, i.e. patients who are admitted to the hospital the day they are operated receive the first dosage of LMWH at the evening of the operation day, patients who are admitted to the hospital prior to their operation receive the first LMWH dosage at the evening the day before the operation. If regional anesthesia was planned the patients did not receive 12 h prior surgery any LMWH.

At admittance to the hospital all patients got anti-embolism stockings, i.e. all patients have anti-embolism stockings during surgery and for the entire hospital period. The compliance wearing GCS was controlled through ward rounds three times a day by the nurses.

All inpatient cases were generally coded by a trained coding specialist using current coding guidelines (German Diagnosis Related Groups, G-DRG) and diagnoses were defined according to the ICD (International Classification of Diseases). The coding specialists received regular internal and external training and final coding was checked for correctness and completeness by a senior physician. Controls of coding accuracy were checked by internal and external audits. The quality of the medical and nursing coding for 2015 is shown as an example (Appendix C). The database analysis with the secondary diagnosis I26 (pulmonary embolism), I80.2 and I80.3 (thrombosis of the lower extremity) did not distinguish between preoperative and postoperative pulmonary embolisms and thrombosis. For this reason, the complete medical records were specifically examined to exclude patients with preoperative or ‘status at diagnosis’ PE and DVT from the case-control study. Thus, this filter served to include postoperatively diagnosed PE and DVT but to exclude preoperatively existent or ‘status after diagnosis’ from the analysis.

Symptomatic DVT was verified in all cases by duplex sonography or phlebography. PE was verified in all surviving patients by CT. For all deceased patients, the simplified Wells II score (Appendix D) was also determined in accordance with the German S2k guideline for diagnosis and treatment of PE [19,20]. In the cases of fatal PE (n = 12), two patients had previously undergone a radiological diagnosis with confirmed evidence of a PE. In one patient, the diagnosis was made postmortem at autopsy. In all other patients with a fatal PE (n = 9), the diagnosis was made clinically, using the Wells II score of≥2 (mean = 2.3).

Two database requests for the specified period were made. The database analysis included case number, age, gender, date of admission, date of discharge, main ICD diagnosis, main OPS code, secondary ICD diagnosis I26 (PE) and I80.2/I80.3 (DVT) (modified ICPM for Germany).

Risk stratification (Table 1) was performed independently by two investigators (KS, EH) according to the German S3 Guideline and the American ACCP Guideline as a 3-part risk classification of operative risk [3,7]. Discrepancies were resolved by consensus. Individual data are available at mendeley.data (https://doi.org/10.17632/ys2vw45pkf.3).

**Table 1:** Risk stratification for venous thromboembolism (VTE).

Low Risk	Upper extremity surgery, soft tissue surgery, inguinal hernia, appendectomy, cholecystectomy
Moderate Risk	Lower limb surgery, large incisional hernias, small and large bowel resection, hiatal hernias
High Risk	Knee and hip replacement. Hepatic, gastric, esophageal, rectal, pelvic surgery

Risk classification corresponding to operative procedure, according to the German S3-guideline, derived from the American guideline American College of Chest Physicians (ACCP 2004).

### 2.2 Setting

The Asklepios Klinik Langen is an academic teaching hospital of the Goethe-University Frankfurt and part of the Asklepios Group, comprising 150 clinics and health care facilities in Germany (https://www.asklepios.com/en/). In Langen 406 planned beds are available. Every year 15 000 inpatients are treated and more than 7000 operations in the fields of ophthalmology, gynecology and obstetrics, ENT, neurosurgery, orthopedics/trauma surgery (trauma center of the German Society for Trauma Surgery/DGU) and general, visceral and thoracic surgery (certified competency center for minimally invasive surgery by the Surgical Association for Minimally Invasive Surgery of the German Society of General and Visceral Surgery - CAMIC) are carried out. The clinic is also incorporated into the surgical study network CHIRNet (http://chir-net.de/) a regional study center.

### 2.3 Statistical methods

To increase the comparability of the two therapy groups and come as close as possible to a randomized parallel-group design, propensity matching was implemented [21]. By author-consensus, a propensity score was calculated for each patient using logistic regression based on age and gender demographic variables and a tri-level variable for the at-risk group. A 1:1 ″nearest neighbor matching” was performed for the two therapy groups. A caliper of 0.1 standard deviations of the log of the propensity score was chosen and exact matching was performed for the two categorical variables, gender and risk group. This matching resulted in 11 312 patients in one therapy group being assigned a matching partner in the other therapy group so that the propensity score difference for the couples was minimized.

For this sample of 22 624 patients, descriptive representations were drawn up for sample description and comparison of the two therapy groups - mean and standard deviation for age and absolute and relative frequencies for the nominally scaled variables, gender and risk group. To ratify the comparability of the two groups, an exploratory significance test (*t*-test) for age was performed. Gender and risk group corresponded exactly.

The primary target parameter was PE occurrence. Differences between therapy groups in terms of this outcome variable were analyzed using crosstabs and Fisher's exact test. RR (relative risk) was determined, with an asymptotic confidence interval of 95% (CI 95%). In addition, the joint quota ratio was estimated according to Mantel-Haenszel, after determining the odds ratios. SPSS from IBM in version 24 was employed as the statistics program.

Effect sizes for continuous data (age, length of stay) were assessed. Different tools were applicable like Cohen's d, Hedges's g and Glass's Δ. We used Cohen's d because the standard derivation did not differ significantly between the groups and the sample size was larger than 20. As such d from 0.2 is considered to reflect a small effect size, d from 0.5 indicates a moderate effect size and d from 0.8 indicates a large effect size.

## 3 Results

### 3.1 Participants

A total of 24 273 patients were included: 11 010 men and 13 263 women. Of the patients enrolled, 11 661 wore GCS, while 12 612 wore no GCS. Age in the two groups was not significantly different (p = 0.478). Before matching, the groups differed significantly in terms of gender and risk score (p = 0.0005) (Table 2).

**Table 2:** Total sample (n = 24 273) prior to propensity matching – clinical characteristics.

	LMWH alone	LMWH + GCS	P
Age in years; mean (SD)	61.8 (18.7)	61.6 (18.4)	0.478
Gender; f/m (%)	6692/5920 (53.1%/46.9%)	6571/5090 (56.4%/43.6%)	<0.0005
Low Risk; n (%)	8521 (67.6)	7962 (68.3)	<0.0005
Moderate Risk; n (%)	2239 (17.8)	2225 (19.1)	
High Risk; n (%)	1852 (14.7)	1474 (12.6)	

LMWH: Low Molecular Weight Heparin.

GCS: Graduated Compression Stocking.

SD: standard deviation.

In the propensity-matched group, the total sample of this large number of patients (n = 22 624) differed significantly in terms of age (p = 0.0005). However, the Cohen's value (d), with a value of 0.07, was below the threshold for a low effect. The groups did not significantly differ (p = 1) with regard to the target parameters, gender and risk scores (Table 3).

**Table 3:** Propensity matched pairs (n = 22 624) – clinical characteristics.

	LMWH alone	LMWH + GCS	p	Cohen's d
Age in years; mean (SD)	62.9 (18.3)	61.6 (18.4)	<0.0005	0.07
Gender; f/m (%)	6291/5021 (55.6/44.4)	6291/5021 (55.6%/44.4)	1	
Low Risk; n (%)	7731 (68.3)	7731 (68.3)	1	
Moderate Risk; n (%)	2114 (18.7)	2114 (18.7)		
High Risk; n (%)	1467 (13.0)	1467 (13.0)		

LMWH: Low Molecular Weight Heparin.

GCS: Graduated Compression Stocking.

SD: standard derivation.

After propensity score matching the length of stay (LOS) was 8.95 ± SD 10.71 days with LMWH + GCS; 8.48 ± SD 11.79 LMWH alone. Due to the high number of patients, this is significantly different. However, Cohen's d = 0.042, which is indicating an irrelevant effect size.

### 3.2 Main results

The relative risk for a PE without GCS application was 0.795 [CI95% 0.632–1.000] for the whole population, before propensity matching. Relative risks in the subgroups are as follows: Low Risk RR 0.773 [CI 95% 0.528–1.130]; Moderate Risk RR 0.997 [CI 95% 0.656–1.516]; High Risk RR 0.553 [CI 95% 0.404–0.775]; (p > 0.05). A fatal PE occurred in 4 out of 19 patients who did not use GCS and in 8 out of 29 patients using GCS, at a relative risk of 0.763 [CI95% 0.267–2.184] (p > 0.05) (Table 4).

**Table 4:** Symptomatic or Fatal Pulmonary embolism in total sample (n = 24 273) prior to propensity matching - Relative Risk [95% CI].

		LMWH alone	LMWH + GCS	RR (p > 0.05)	CI95%
Pulmonary embolism	Low Risk; n (%)	6 (0.1)	10 (0.1)	0.773	[0.528–1.130]
	fatal; n (%)	1 (17)	2 (20)	0.833	[0.095–7.347]
	Moderate Risk; n (%)	11 (0.5)	11 (0.5)	0.997	[0.656–1.516]
	fatal; n (%)	3 (27)	2 (18)	1.500	[0.308–7.297]
	High Risk; n (%)	2 (0.1)	8 (0.5)	0.553	[0.404–0.775]
	fatal; n (%)	0 (0)	4 (50)	–	–
Total PE; n (%)		19 (0.2)	29 (0.2)	0.795	[0.632–1.000]
Fatal PE; n (%) [Fatal PE/Total PE]		4 (21)	8 (27)	0.763	[0.267–2.184]

LMWH: Low Molecular Weight Heparin.

GCS: Graduated Compression Stocking.

PE: Pulmonary Embolism.

p: Significance level using Fisher's exact test.

RR: Relative Risk.

CI 95%: Asymptotic 95% Confidence Interval.

The relative risk for a DVT without GCS application was 0.715 [CI95% 0.380–1.345] (p > 0.05) for the whole population (Table 5).

**Table 5:** Symptomatic DVT in total sample (n = 24 273) prior to propensity matching - Relative Risk [95% CI].

		LMWH alone	LMWH + GCS	RR (p > 0.05)	CI95%
DVT	Low Risk; n (%)	3 (0.04)	5 (0.06)	0.560	[0.134–2.345]
	PE; n	1	1	1.667	[0.155–17.895]
	Moderate Risk; n (%)	6 (0.27)	4 (0.18)	1.491	[0.421–5.275]
	PE; n	2	1	1.333	[0.173–10.255]
	High Risk; n (%)	8 (0.43)	13 (0.88)	0.490	[0.204–1.179]
	PE; n	1	0	0.467	[0.213–102.476]
Total DVT; n (%)		17 (0.1)	22 (0.19)	0.715	[0.380–1.345]
PE; n		4	2	2.588	[0.536–12.503]

LMWH: Low Molecular Weight Heparin.

GCS: Graduated Compression Stocking.

DVT: Deep Vein Thrombosis.

PE: Pulmonary Embolism (count of patients having DVT simultaneously).

p: Significance level using Fisher's exact test.

RR: Relative Risk.

CI 95%: Asymptotic 95% Confidence Interval.

After matching by propensity score analysis, the relative risk of the total collective was 0.999 [CI 95% 0.998–1.00]. The subgroups showed similar relative risk: Low Risk RR 0.999 [CI 95% 0.998–1.000]; Moderate Risk RR 0.999 [CI95% 0.995–1.003]; High Risk RR 0.996 [CI95% 0.992–1.000] (p > 0.05). A fatal PE occurred in 4 out of 16 patients who did not use GCS and in 8 out of 29 patients using GCS. RR was 0.906 [CI95% 0.322–2.547] (p > 0.05) (Table 6).

**Table 6:** Propensity matched pairs (n = 22 624) – symptomatic or fatal pulmonary embolism.

		LMWH alone	LMWH + GCS	RR (p > 0.05)	CI95%
Pulmonary embolism	Low Risk; n (%)	6 (0.1)	10 (0.1)	0.999	[0.998–1.000]
	fatal; n (%)	1 (17)	2 (20)	0.833	[0.095–7.347]
	Moderate Risk; n (%)	8 (0.4)	11 (0.5)	0.999	[0.995–1.003]
	fatal; n (%)	3 (38)	2 (18)	2.063	[0.442–9.621]
	High Risk; n (%)	2 (0.1)	8 (0.5)	0.996	[0.992–1.000]
	fatal; n (%)	0 (0)	4 (50)	–	–
Total PE; n (%)		16 (0.1)	29 (0.3)	0.999	[0.998–1.000]
Fatal PE; n (%) [Fatal PE/Total PE]		4 (25)	8 (27)	0.906	[0.322–2.547]

LMWH: Low Molecular Weight Heparin.

GCS: Graduated Compression Stocking.

PE: Pulmonary Embolism.

p: Significance level using Fisher's exact test.

RR: Relative Risk.

CI 95%: Asymptotic 95% Confidence Interval.

## 4 Discussion

The results of the present study support a critical view of potential benefit from general prophylactic use of antithrombotic stockings, added to LMWH [[11], [12], [13], [14]]. Kröger et al. see no general indication for the use of GCS without evidence and considering possible side effects [11]. The study by Cohen et al. and the CLOTS-1 Trial also report no positive effect in preventing thromboembolic events by using GCS [15,16]. A positive attitude towards the use of GCS, as in the Cochrane analysis is largely based on older data with small patient numbers [29]. The Edoxaban-Approval Study saw a reduction in the VTE rate from 13% to 6%, using GCS [30]. However, there seems to be variation in the prophylactic effect of GCS, when using new oral anticoagulants, compared to LMWH.

The analysis of Fuji et al. of the Phase 3 Edoxaban study in Total Knee Arthroplasty describes post hoc the benefit of GCS when all VTEs (asymptomatic thrombosis, symptomatic thrombosis and pulmonary embolism) were included [31]. A total of 201 patients, divided into four groups, GCS with edoxaban or enoxaparin versus no GCS with edoxaban or enoxaparin were compared. The authors concluded: “Although the incidence of VTE was >2-fold lower among patients receiving anticoagulation plus GCS compared with those receiving anticoagulation alone, statistical significance was not achieved.” Mandavia et al. investigated the benefit of GCS in a meta-analysis including a total of 27 RCTs [12]. For this purpose, 12 481 patients without GCS application and 1292 patients with GCS application were included in the analysis. The authors concluded that a possible advantage of the application of GCS could not be confirmed with the existing data. Arabi et al. found out in a randomized controlled trial with critically ill patients (n = 2003) that an adjunctive intermittent pneumatic compression did not result in a significantly lower incidence of DVT or PE [32]. In the systematic review of Milinis et al. there is insufficient evidence to recommend GCS in conjunction with extended pharmacological prophylaxis in patients undergoing orthopedic and abdominal surgery [33]. A total of 9824 patient in 16 studies treated with extended pharmacological thromboprophylaxis in 0.2% a PE occurred. In three studies with a total of 337 patients having GCS as an adjunct to pharmacological thromboprophylaxis no PE (0%) was reported. The GAPS study (total number of patients n = 1905) (Graduated compression stockings as adjuvant to pharmaco-thromboprophylaxis in elective surgical patients: randomized controlled trial) showed that LMWH alone is non-inferior to LMWH plus GCS [34]. In this report it was shown that the incidence within 90 days for symptomatic PE in a moderate and high-risk population (LMWH alone: 2/937 and LMWH + GCS 1/921) was as low as in our study population. The symptomatic DVT-rate (LMWH alone: 2/937 and LMWH + GCS: 1/921) was as low as in our population, respectively and thus fairly comparable with our data.

### 4.1 Limitations

To prove a hypothesis, the highest informative value is achieved with a prospective, randomized, controlled multicenter study [22]. The present study design, i.e. case-control study, described as quasi-experimental, comes to bear. This is an “uncontrolled before and after study” with a change being measured before and after an intervention. An inherent risk lies in not detecting interfering factors [23]. Propensity matching, however, can be used to adjust for known disturbances, and bring the result-quality close to that of a randomized study [24,25]. Unknown factors can influence randomized trials, which in a study period of 10 years, as presently was the case, cannot be excluded. Co-medication or improved physiotherapy and mobilization strategy in the context of a fast track concept, for example, could be confounding factors. Formally, we have not implemented an ERAS protocol, but our priorities are of course set to early mobilization and enteral nutritional support of patients for the whole study time. In addition, during the period under review, there were no strategic changes in operative decision making and treatment processes did not fundamentally change.

Our analysis regarding open vs laparoscopic procedures reveals that there are more procedures performed laparoscopically in the second period without GCS: 1553 (with GCS) vs 1919 (without GCS). In the risk score III group (see Table 1) the numbers are for laparoscopic rectal resections 22 (LMWH + GCS) vs 76 (LMWH alone). Due to the high total number of patients, we argue that this difference has no influence upon the incidence of PE or DVT in our study; Moreover, the meta-analysis of Cui et al. does not find a difference of PE when comparing open and laparoscopic colorectal surgery [26].

The total sample of this large number of patients (n = 22 624) differed in the propensity score matched analysis significantly in terms of age (p = 0.0005) and length of stay. However, Cohen's value (d) was below the threshold for a low effect.

Another vulnerability associated with the present study is its retrospective nature, whereby data quality could be criticized. However, since continuous auditing of the medical records regularly demonstrated high quality in medical and nursing documentation, we consider the data to be valid. Other limitations of the study are that we cannot comment on complications of antithrombotic stockings/LMWH therapy and that we report only in-hospital PE rates, though PE may also occur post-hospital [27]. It may be argued that our symptomatic or fatal postoperative PE rate and symptomatic DVT rate could be too low as we focus on in-hospital data since it is known that PE and DVT can occur of course after the discharge of the patient. On the other hand, our hospital is well networked with registered doctors with their practice who minister the postoperative care outside the hospital. Thus, we are informed about relevant complications that occur beyond the hospital stay. This exchange of information also takes place at regular meetings participated by our surgeons and the practitioners. Therefore, in our experience complication rates (eg PE) after discharge do not increase significantly. Supporting this view – The GAPS trial takes also into account out-of-hospital symptomatic PE and DVT event rates which are in the same range of our results.

The influence of the GCS material quality, with regard to reducing pulmonary embolisms and deep venous thrombosis, cannot be criticized since the stockings had a good pressure profile compared to other materials in technical ex vivo testing [28].

## 5 Conclusion

In our population we found lower PE rates compared to older historical controls. Our PE rates do not differ from actual data, like the GAPS trial. There was no increase in the relative risk of PE in the group not using GCS compared to the group using GCS additionally to LMWH-prophylaxis, both in the overall population and in the high risk group. Thus, the GAPS prospectively randomized trial data are externally validated in a real world scenario.

## Ethical approval

Ethical approval was acquired from by the Ethics Committee of the Hessian State Medical Association (FF 117-2016).

## Sources of funding

This research did not receive any specific grant from funding agencies in the public, commercial, or not-for-profit sectors.

## Author contribution

All authors have made substantial contributions to conception and design, or acquisition of data, or analysis and interpretation of data; drafted the submitted article or revised it critically for important intellectual content; provided final approval of the version to be published; and have agreed to be accountable for all aspects of the work in ensuring that questions related to the accuracy or integrity of any part of the work are appropriately investigated and resolved.

## Consent

Because of the retrospective character of the study and the anonymous data written informed consent was not necessary.

## Research registration unique identifying number (UIN)

Name of the registry: German Registry of clinical trials (DRKS).

Unique Identifying number or registration ID: DRKS00015507.

Hyperlink to your specific registration (must be publicly accessible and will be checked):

https://www.drks.de/drks_web/

## Guarantor

K.Suna, E.Hanisch and A.Buia guarantee that the reported data are correct and genuine. All authors had access to the data. K. Suna, E.Hanisch and A.Buia are the Guarantor for this trial and controlled the decision to publish.

## Provenance and peer review

Not commissioned, externally peer reviewed.

## Declaration of competing interest

All authors have no conflict of interest to declare.

## Figures and Tables

**Table:** 

	German S3-Guideline 2015	NICE Guideline 2018	American College of Chest Physicians Evidence-Based Clinical Practice Guidelines 2012	VTE Risk
Recommendation/evidence level		none/very low	no/Grade 2C	very low
	may/low	consider/very low - low	may/Grade 2C	Low
	may/low	consider IPC/very low-low	may/Grade 2C	Moderate
	may/low	offer IPC/very low-low	may/Grade 2C	High
		consider/offer IPC/GCS/very low - low	Intermittent pneumatic compression/grade 1C	Total hip arthroplasty/total knee arthroplasty/hip fracture surgery

**Table:** 

Structural criteria
**Cooperation:** • Physicians • Physiotherapists
**Qualification/Responsibility:** • Licensed nursing personnel (GKP) • Nursing personnel in 1st training year (GKPS) - Implementation of planned prophylactic measures • Nursing assistants (KPH) **Nursing staff is responsible, according to qualification** • To detect thrombosis risk • To plan and perform interventions to support venous return • To pass on information to all involved professional groups • To document all measures and observations performed on patients **Nursing staff possesses up-to-date knowledge and the competence to** • Assess thrombosis risk • Choose appropriate aid
• Plan and implement effective thrombosis prophylaxis • Evaluate chosen interventions • Guide and counsel patient and family to promote implementation
Process criteria
**Process description:** • Initial assessment of thrombosis risk for each patient, taking following risk factors into account: - limited mobility (immobility, paralysis, cast) - pain-related restrictive posture - cardiac insufficiency - chronic venous insufficiency - increased intravascular coagulation tendency (dehydration, blood loss, pathol. cell propagation, medication; for example, cortisone, contraceptives, cytostatic agents) - vessel wall damage (traumatic, for example OP, degenerative or inflammatory) - coagulation disorder - overweight (BMI > 25) - pregnancy, postpartum - malignant disease - previous thromboses or embolisms - infections - age > 65 years - smoking • The Frowein DVT score can be used to aid risk-estimation, if necessary
**Basic principals:** • when a hazard has been identified: suitable interventions, implementation and intervals must be documented • inform patient about all measures • observe hygienic aspects

**Table:** 

	Jan 15	Feb 15	Mar 15	Apr 15	May 15	Jun 15	Jul 15	Aug 15	Sep 15	Oct 15	Nov 15	Dec 15
**1**	**84**	**99**	**68**	**90**	**90**	**90**	**86**	**88**	**81**	**83**	**90**	**89**
**2**	**99**	**100**	**98**	**100**	**100**	**100**	**80**	**100**	**98**	**74**	**94**	**100**
**3**	**90**	**100**	**100**	**93**	**92**	**98**	**100**	**100**	**100**	**93**	**100**	**100**
**4**	**100**	**100**	**60**	**100**	**100**	**100**	**80**	**100**	**100**	**80**	**40**	**40**
**5**	**90**	**100**	**100**	**85**	**100**	**100**	**100**	**100**	**100**	**100**	**100**	**100**
**6**	**92**	**100**	**98**	**86**	**98**	**100**	**100**	**100**	**100**	**100**	**100**	**100**
**7**	**96**	**100**	**90**	**94**	**94**	**88**	**94**	**92**	**100**	**100**	**100**	**100**
**8**	**100**	**80**	**85**	**100**	**100**	**98**	**80**	**85**	**65**	**65**	**65**	**80**
**9**	**100**	**100**	**100**	**100**	**100**	**100**	**100**	**100**	**100**	**100**	**100**	**100**
**Total:**	**94**	**98**	**89**	**95**	**96**	**96**	**90**	**95**	**93**	**87**	**91**	**93**

1 = Medical anamnesis and medical findings 2 = Nursing anamnesis 3 = Care planning 4 = Recording of nurses' relevant secondary diagnoses 5 = Medical prescription 6 = Day chart 7 = Clinical course documentation/nursing report 8 = Discharge and relocation documentation 9 = Formal.

**Table:** 

Variable	Points
Clinical signs or symptoms of deep-vein thrombosis	1
Alternative diagnosis less likely than pulmonary embolism	1
Heart rate >100 beats/min	1
Immobilization or surgery in the previous 4 weeks	1
Previous venous thromboembolism	1
Hemoptysis	1
Active cancer	1

A total score≥2 indicates that pulmonary embolism is likely.

## Appendix A Guidelines for use of Graduated Compression Stockings

Classification according to the German S3-Guideline:

1. Highly recommended: high level of evidence - systematic review (metaanalysis) or RCT (therapy) or cohort study (risk factors, diagnostics)
2. Should be recommended: middling level of evidence - RCT or cohort study of limited quality
3. May be recommended: low level of evidence - RCT or cohort study of poor quality, all other study designs, expert opinion

The NICE guideline is a GRADE scheme:

1. High: Further research is very unlikely to change our confidence in the estimate of effect
2. Moderate: Further research is likely to have an important impact on our confidence in the estimate of effect and may change the estimate
3. Low: Further research is very likely to have an important impact on our confidence in the estimate of effect and is likely to change the estimate
4. Very low: Any estimate of effect is very uncertain

The ACCP Guideline is precisely differentiated:

1. 1A strong recommendation, high quality evidence: RCTs without important limitations or overwhelming evidence from observational studies
2. 1B strong recommendation, moderate quality evidence: RCTs with important limitations (inconsistent results, methodological flaws, indirect, or imprecise) or exceptionally strong evidence from observational studies
3. 1C strong recommendation, low-quality or very low-quality evidence: Observational studies or case series
4. 2A weak recommendation, high-quality evidence: RCTs without important limitations or overwhelming evidence from observational studies
5. 2B weak recommendation, moderate-quality evidence: RCTs with important limitations (inconsistent results, methodological flaws, indirect, or imprecise) or exceptionally strong evidence from observational studies
6. 2C weak recommendation, low-quality or very low-quality evidence: Observational studies or case series

## Appendix B SOP Thrombosis prophylaxis - care

Goal:

• Maintenance and promotion of venous return
• Prevention of vessel wall damage
• Reduce intravascular coagulation disposition

Definition:

• Nursing interventions to ensure venous return

Indications for use of standards:

• Increased thrombosis risk due to existing risk factors (see process criteria)
• Diminished venous return
• Vessel wall damage
• Increased intravascular coagulation tendency

**Contraindications:** none **Note:** Thrombosis risk can be determined using the Frowein DVT score.
